# Establishment and application of risk classification model for lead in vegetables based on spectral clustering algorithms

**DOI:** 10.1002/fsn3.2718

**Published:** 2022-01-19

**Authors:** Tong‐qiang Jiang, Zheng Wang, Qing‐chuan Zhang, Zu‐zheng Wang, Bao‐lian Cheng

**Affiliations:** ^1^ National Engineering Laboratory for Agri‐product Quality Traceability Beijing Technology and Business University Beijing China; ^2^ Nanjing Tech University Nanjing China; ^3^ Beijing Technology and Business University Beijing China

**Keywords:** lead, risk assessment, risk classification, spectral clustering algorithms, vegetables

## Abstract

This study aims to evaluate the risk of lead pollution in 9 kinds of vegetables consumed by residents in 20 provinces/cities of China. Sampling data and vegetable consumption data from 20 provinces/cities in 2019 were used. Combined with dietary exposure assessment, the vegetable categories and provinces were paired, and a risk classification model based on spectral clustering algorithms was proposed. The results of the spectral clustering algorithm showed that the risk level of lead pollution in vegetables can be divided into five levels. The combination of vegetable‐province/cities at the risk level of 1 and 2 accounted for 92.78%, and that at the risk level of 4 and 5 accounted for 2.22%. The high‐risk combinations were fresh edible fungus–Shaanxi, fresh edible fungus–Sichuan, and fresh edible fungus–Shanghai and bean sprouts–Guangdong. In the proposed model, objective data were used as the classification index, and the spectral clustering algorithm was employed to select the optimal risk classification in a data‐driven way. As a result, the influence of subjective factors was effectively reduced, the risk of lead pollution in vegetables was classified, and the results were scientific and accurate. This study provides a scientific basis of supervision priorities for regulatory departments.

## Graphical abstract

The high‐risk combinations were fresh edible fungus‐Shaanxi, fresh edible fungus‐Sichuan and fresh edible fungus‐Shanghai and bean sprouts‐Guangdong. The high‐risk combinations mainly indicated the priority of attention.

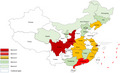

## 1 INTRODUCTION

Lead is a nonessential metal element for plants (Naiming, 2013). When lead is absorbed by plants and accumulated to a certain extent, crop quality can be affected (Jinda et al., 2005). Once lead enters the body through consumption, it is harmful to human health (Honghong et al., 2020). Low concentrations of lead damages the human nervous system and kidney, and high concentrations of the lead causes cancer or death. A large number of studies have proved that lead is one of the important pollutants affecting food safety and human health (Kaiser, 1998; Markus & McBratney, 2001).

Vegetables are an important part of the human diet and one of the main sources of minerals and vitamins required by humans (Grusak & DellaPenna, 1999; Welch & Graham, 1999). With the improvement of living standards, the demand for pollution‐free vegetables in China is increasing day by day.

In recent years, lead pollution in crops and its health risk to the human body has been highlighted in research (Rongguang et al., 2010; Zhou et al., 2020). Tuanhui et al. (2019) assessed the health risk of crops around a mining area in Fujian Province through the target hazard factor (THQ) of the health evaluation model. It is found that among the crops in the mining area, lead pollution is the second major pollution of the root, potato, and leaf vegetables, and there are compound health risks for crops in the mining area of Fujian Province. Juan et al. (2021) evaluated the health risk of lead in cultivated crops in the eastern regions of Yunnan Province. The results showed that there is no obvious carcinogenic or noncarcinogenic risk of lead in farmland soil of eastern Yunnan to residents, and the noncarcinogenic risk of lead for children is greater than that for adults in different regions. In the existing studies, the risk assessment of heavy metals in crops is mostly based on point assessment in certain areas, and there is a small amount of data and nationwide studies.

In terms of risk classification models, various qualitative, semi‐quantitative, and quantitative models have been developed. For example, the iResk model is established by the U.S. Food and Drug Administration (FDA); the health risk classification of chemicals in food is proposed by the China National Center for Food Safety Risk Assessment (CFSA) (Zhou et al., 2014), and the semi‐quantitative risk classification method is put forward by Guangdong Center for Disease Control and Prevention (Chen et al., 2013). The iResk model of FDA is a quantitative model applicable to both microorganisms and chemicals. In this model, the Monte Carlo simulation technology, disease burden estimation, and other statistical methods are used with mathematical functions to integrate and calculate multivariable data such as hazards, consumption, and dose–response relationship, so as to complete risk assessment, comparison, and sequencing of food hazard combination from primary production to consumption (Chen et al., 2013). This quantitative model has a wide application range, but it is complex and indigestible, requiring a large number of computer operations. The UK Veterinary Residues Committee (VRC) addresses veterinary drug management and proposes a classification model. In this model, the risk matrix and scoring method are adopted, and six indexes are set for scoring calculation: hazard nature, hazard intensity, the proportion of drug animals in diet, drug frequency, highly exposed population, and detection of drug residues; finally, the risk level is obtained by scores (VRC, 2010). This model is relatively simple and convenient, and achieves a better balance in accuracy and efficiency. However, these indexes have strong pertinence to veterinary drugs and are not widely applicable. Aiming at the risk classification of food hazard factors, the above models have different scopes of application, simplicity, and index setting due to different regulatory needs.

According to the statistics released by the State Administration for Market Regulation, about 200,000 sampling data of lead contamination in food are generated every year. To this end, a food safety risk classification model based on the sampling data was established in this study. The national sampling data of lead in vegetables in 2019 and the vegetable consumption data were employed, the dietary exposure assessment was used to calculate the lead dietary intake of residents in 20 provinces/cities of China. Finally, the spectral clustering algorithm was employed to determine the risk level of lead pollution in vegetables. This model can provide a quantitative basis for decision‐making and supervision priorities by the sampling data and risk level.

## 2 MATERIAL AND METHODS

### 2.1 Data sources

The lead pollution data of vegetables in this study came from the National Food Safety Sampling Data in 2019, with a total of 11,456 samples. The data of vegetable consumption of residents in 20 provinces/cities were from the fifth Total Diet Study. The method of stratified, multistage cluster random sampling proportional to the population was adopted in the fifth Total Diet Study, and a dietary questionnaire survey on the main food consumed by residents in 20 provinces/cities of China was conducted. In our study, vegetables were divided into nine categories: leafy vegetables, root and potato vegetables, legume vegetables, bean sprouts, melon vegetables, bulb vegetables, Brassica vegetables, solanaceous vegetables, and fresh edible fungus. Toxicological data were obtained from reports or bibliographic retrieval of international organizations such as the Joint Expert Committee on Food Additives of the United Nations Food and Agriculture Organization, the World Health Organization (FAO/WHO JECFA), and the United States Environmental Protection Agency (US EPA). The reference dose (RfD) of lead is 3.5 μg/(kg d), the lower limit of 95% confidence interval for 1% reference dose (BMDL_01_) is 0.6 μg/kg bw per day.

### 2.2 Methods

#### 2.2.1 Model building

According to the risk assessment method and the model purposes, the main influencing factors of health risks caused by food pollutants were considered, and Nemerow integrated pollution index (NIPI), hazard index (HI), and margin exposure (MOE) were selected as the indexes of the model. The average content, median (P50), 95th quantile (P95), and maximum value of lead in vegetables were selected as the characteristics of food pollution to calculate the exposure of lead under different pollution levels.

The NIPI reflects the characteristics of food pollution. This index has been used to evaluate the heavy metal pollution in the air (Bekhet & Yasmin, 2013), water (Zang et al., 2017), vegetables (Li et al., 2013; Sawut et al., 2018; Shengbang & Baixi, 2015), rice (Junxiao et al., 2018), and soil (Han et al., 2018; Mazurek et al., 2017). According to the sampling data in different provinces/cities, the lead pollution degree of sampling data is calculated by using the NIPI as follows:

$$P_{i,j}=\frac{X_{i,j}}{S_{j}}$$

where $P_{i,j}$ is the pollution index of vegetable j in province/city i; $X_{i,j}$ is the detection value of lead content in vegetable j in province/city i (mg/kg); $S_{j}$ is the national limit standard of lead in vegetable j (mg/kg).

$$P_{c(i.j)}=\sqrt{\frac{P_{\max(i,j)}^{2}+P_{ave(i,j)}^{2}}{2}}$$

where $P_{c(i,j)}$ is the NIPI of vegetable j in province/city i; $P_{\max(i,j)}$ is the maximum pollution index of vegetable j in province/city i; $P_{\text{ave}(i,j)}$ is the average value of pollution index $P_{i,j}$ of vegetable j in province/city i.

The HI is used to characterize the noncarcinogenic risk of lead in vegetables by lead exposure and reference dose. The expression is as follows:

$${\text{HI}}_{i,j}=\frac{{\text{EDI}}_{i,j}^{95}}{\mathrm{RfD}}$$

$${\text{EDI}}_{i,j}^{95}=\frac{{\text{FC}}_{i,j}\times X_{i,j}^{95}}{W}$$

where $HI_{i,j}$ is the lead noncarcinogenic risk of vegetable j in province/city i; RfD is the oral reference dose of lead (μg/kg d); ${\text{EDI}}_{i,j}^{95}$ (estimated daily intake) is the estimated daily intake of lead through vegetable j in the province/city i under high exposure (P95); ${\text{FC}}_{i,j}$ is the average daily consumption of vegetable j in province/city i (kg/d); $X_{i,j}^{95}$ is the 95th quantile (mg/kg) of lead sampling content in vegetable j in province/city i; W is the average body mass of residents (60 kg).

The MOE is used to characterize the chronic dietary intake risk of lead by the lower limit of 95% confidence interval of 1% benchmark dose of lead and the exposure. The expression is as follows:

$${\text{MOE}}_{i,j}=\frac{{\text{BMDL}}_{01}}{{\text{EDI}}_{i,j}^{50}}$$

$${\text{EDI}}_{i,j}^{50}=\frac{{\text{FC}}_{i,j}\times X_{i,j}^{50}}{W}$$

where $MOE_{i,j}$ is the risk of chronic dietary intake of lead in vegetable j in province/city i; ${\text{EDI}}_{i,j}^{50}$(estimated daily intake) is the estimated daily intake of lead from vegetable j in province/city i under moderate exposure (P50); ${\text{FC}}_{i,j}$ is the daily consumption of vegetable j in province/city i (kg/d); $X_{i,j}^{50}$ is the median lead content of vegetable j in province/city i (mg/kg); W is the average body mass of residents (60 kg).

##### Computational environment

In this study, the Windows10 64 system was used as the experimental environment of spectral clustering algorithm; the Intel(R) Xeon(R) E5‐1620 v4 @3.50GHz was used as processor; the running memory was 64GB; the NVIDIA GTX 1060 Ti was used as the data accelerator, and Python and related libraries were used as the experimental programming language.

##### Clustering classification

The possibility of excessive pollutants, exposure, and harmfulness of food pollutants were considered to quantify the risk factors of pollutants, and the food safety risk assessment model was established by the above three indexes. Clustering is a process of dividing a given sample into multiple clusters to obtain samples in the same cluster with high similarities and different clusters with low similarity. Clustering analysis can be used to mine deep information of data. With a low sensitivity to sample shape and good support for high‐dimensional data, the spectral clustering algorithm can achieve good clustering performance in the arbitrary shape of sample space and is suitable for analyzing the model data of this study.

Scientific and accurate determination of classification level is one of the main problems of food safety risk classification. In this study, the clustering algorithm was used to determine the risk level of food pollutants. Through calculating the Calinski–Harabasz (CH) index of different parameters‐cluster number combination (the larger the CH index, the smaller the total similarity between clusters, and the better the clustering effect), the spectral clustering algorithm was used to select the optimal parameter and cluster number combination, carry out risk classification in a data‐driven way, and eliminate the subjectivity of risk classification.

The main process of spectral clustering algorithm was as follows:

1. A matrix W describing the characteristics of the sample was constructed from the data sample.
2. The eigenvalues and eigenvectors of matrix W were calculated and sorted.
3. The eigenvectors corresponding to the first k eigenvalues after sorting were taken, and the vectors were arranged according to the column direction to form a new solution space.
4. The K‐means clustering was adopted in the new solution space, and finally, the clustering results were mapped to the original solution space.

##### Data processing

According to the principle of *Low levels of pollutants credible evaluation* proposed at the second meeting of WHO Global Environmental Monitoring System/Food (GEMS/FOOD), when the proportion of undetected data was less than 60%, all undetected data shall be replaced by 1/2 of the limit of detection (LOD); when the proportion of undetected data was higher than 60%, all undetected data are replaced by LOD (Xuqing et al., 2002). In this study, the undetected data were given 1/2 LOD value for statistical calculation.

## 3 RESULTS

### 3.1 Lead pollution in vegetables

As shown in Table 1, the vegetables with the highest average lead content were fresh edible fungus in Shaanxi Province (0.0662 mg/kg), followed by fresh edible fungus in Shanghai City (0.0656 mg/kg), and root and potato vegetables in Sichuan Province (0.06 mg/kg).

**TABLE 1:** Average content of lead in different kinds of vegetables in provinces/cities (mg/kg)

Vegetable categories	Shanghai	Inner Mongolia	Beijing	Jilin	Sichuan	Ningxia	Guangdong	Guangxi	Jiangsu	Jiangxi
Leafy	0.025	0.030	0.027	0.020	0.044	0.023	0.023	0.024	0.023	0.027
Root and potato	0.020	0.032	0.020	0.028	0.060	0.020	0.025	0.024	0.022	0.023
Melon	0.035	0.035	0.030	0.030	0.033	0.030	0.030	0.030	0.029	0.030
Brassica	0.022	0.025	0.020	0.020	0.020	0.022	0.025	0.020	0.021	0.031
Solanaceous	0.021	0.025	0.020	0.020	0.027	0.022	0.021	0.020	0.021	0.024
Legume	0.020	0.028	0.022	0.020	0.036	0.025	0.056	0.022	0.025	0.029
Bean sprouts	0.022	0.023	0.021	0.021	0.033	0.023	0.030	0.020	0.022	0.025
Fresh edible fungus	0.066	0.031	0.026	0.023	0.052	0.020	0.026	0.020	0.032	0.023
Bulb	0.023	0.045	0.033	0.020	0.022	0.024	0.026	0.023	0.020	0.025

	Hebei	Heilongjiang	Zhejiang	Hubei	Hunan	Fujian	Liaoning	Shaanxi	Qinghai	Henan
Leafy	0.030	0.031	0.025	0.025	0.022	0.031	0.037	0.051	0.023	0.029
Root and potato	0.020	0.024	0.036	0.020	0.020	0.026	0.017	0.032	0.020	0.020
Melon	0.030	0.033	0.020	0.030	0.030	0.030	0.029	0.030	0.022	0.031
Brassica	0.034	0.020	0.019	0.034	0.021	0.021	0.029	0.029	0.016	0.022
Solanaceous	0.024	0.023	0.017	0.021	0.020	0.021	0.022	0.032	0.012	0.022
Legume	0.025	0.029	0.015	0.020	0.023	0.021	0.023	0.020	0.020	0.021
Bean sprouts	0.022	0.023	0.018	0.021	0.023	0.020	0.020	0.022	0.019	0.020
Fresh edible fungus	0.023	0.027	0.027	0.022	0.021	0.020	0.040	0.066	0.017	0.038
Bulb	0.032	0.024	0.024	0.021	0.023	0.023	0.028	0.028	0.027	0.031

China Food Safety Standard specifies the limit index of lead in vegetables, including 0.3 mg/kg for Brassica vegetables and leafy vegetables, 0.2 mg/kg for legume vegetables and 0.1 mg/kg for other vegetables. According to the national standards and Table 2, the fresh edible fungus had the most serious lead pollution, which was excessive in eight provinces/cities (including Beijing and Shanghai); followed by root and potato vegetables in the Jilin, Sichuan, and Fujian Provinces. The lead in bean sprouts in the Guangdong Province and in solanaceous vegetables in the Fujian Province also exceeded the national standard. Among the samples with excessive lead, fresh edible fungus in the Shaanxi and Sichuan Provinces and bean sprouts in the Guangdong Province exceeded the standard most seriously, and the lead content was 4.2 times (0.42 mg/kg), 3.04 times (0.304 mg/kg), and 3.43 times (0.343 mg/kg) of the national standard, respectively.

**TABLE 2:** Maximum value of lead in different kinds of vegetables in provinces/cities (mg/kg)

Vegetable categories	Shanghai	Inner Mongolia	Beijing	Jilin	Sichuan	Ningxia	Guangdong	Guangxi	Jiangsu	Jiangxi
Leafy	0.100	0.104	0.265	0.030	0.250	0.156	0.058	0.290	0.085	0.100
Root and potato	0.020	0.072	0.020	0.110	0.140	0.020	0.030	0.055	0.030	0.030
Melon	0.083	0.082	0.030	0.030	0.087	0.030	0.030	0.030	0.069	0.030
Brassica	0.057	0.030	0.020	0.020	0.020	0.077	0.030	0.020	0.030	0.206
Solanaceous	0.057	0.099	0.020	0.030	0.093	0.082	0.030	0.051	0.060	0.090
Legume	0.020	0.104	0.088	0.030	0.168	0.142	0.343	0.070	0.146	0.090
Bean sprouts	0.070	0.030	0.030	0.030	0.092	0.076	0.030	0.020	0.030	0.070
Fresh edible fungus	0.258	0.085	0.130	0.066	0.304	0.020	0.079	0.020	0.130	0.030
Bulb	0.077	0.094	0.098	0.020	0.079	0.083	0.080	0.083	0.030	0.080

	Hebei	Heilongjiang	Zhejiang	Hubei	Hunan	Fujian	Liaoning	Shaanxi	Qinghai	Henan
Leafy	0.160	0.161	0.093	0.161	0.092	0.182	0.266	0.200	0.140	0.110
Root and potato	0.020	0.062	0.090	0.020	0.020	0.167	0.020	0.080	0.059	0.020
Melon	0.050	0.073	0.070	0.030	0.030	0.030	0.030	0.030	0.057	0.060
Brassica	0.110	0.020	0.080	0.168	0.062	0.084	0.090	0.200	0.091	0.065
Solanaceous	0.092	0.073	0.080	0.030	0.020	0.160	0.093	0.092	0.054	0.069
Legume	0.100	0.067	0.090	0.030	0.077	0.067	0.092	0.020	0.084	0.030
Bean sprouts	0.048	0.056	0.080	0.030	0.057	0.020	0.020	0.061	0.093	0.030
Fresh edible fungus	0.030	0.077	0.189	0.127	0.020	0.020	0.160	0.420	0.045	0.210
Bulb	0.098	0.075	0.090	0.056	0.065	0.087	0.082	0.091	0.066	0.094

### 3.2 Model index results

According to the calculation rules of model indexes, the index values of various vegetables in 20 provinces/cities were obtained, as shown in Table 3.

**TABLE 3:** Index values in the risk assessment model

Place	Legume			Bean sprouts			Root and potato		
	Pc	HI	MOE	Pc	HI	MOE	Pc	HI	MOE
Shanghai	0.100	0.045	3.635	0.519	0.045	3.635	0.200	0.045	3.635
Inner Mongolia	0.381	0.087	7.779	0.269	0.031	7.779	0.560	0.071	7.779
Beijing	0.321	0.053	3.081	0.257	0.053	3.081	0.200	0.053	3.081
Jilin	0.128	0.041	3.926	0.259	0.061	3.926	0.802	0.174	3.926
Sichuan	0.607	0.279	3.651	0.690	0.202	3.651	1.077	0.284	3.651
Ningxia	0.510	0.056	5.099	0.561	0.032	5.099	0.200	0.032	5.099
Guangdong	0.130	0.034	7.096	2.457	0.366	7.096	0.274	0.034	7.096
Guangxi	0.259	0.051	3.179	0.200	0.051	3.179	0.424	0.105	3.179
Jiangsu	0.524	0.062	4.452	0.262	0.055	4.452	0.262	0.050	4.452
Jiangxi	0.334	0.095	6.825	0.526	0.083	6.825	0.265	0.034	6.825
Hebei	0.364	0.074	5.177	0.373	0.047	5.177	0.200	0.031	5.177
Henan	0.129	0.047	5.214	0.256	0.031	5.214	0.200	0.031	5.214
Zhejiang	0.323	0.136	4.392	0.580	0.130	4.392	0.687	0.156	4.392
Hubei	0.128	0.044	3.722	0.259	0.064	3.722	0.200	0.044	3.722
Hunan	0.100	0.057	2.840	0.572	0.092	2.840	0.200	0.057	2.840
Fujian	0.249	0.036	4.525	0.200	0.036	4.525	1.196	0.036	4.525
Liaoning	0.335	0.077	6.352	0.200	0.026	6.352	0.184	0.026	6.352
Shanxi	0.100	0.031	5.200	0.458	0.031	5.200	0.610	0.111	5.200
Qinghai	0.305	0.140	4.391	0.671	0.112	4.391	0.440	0.079	4.391
Heilongjiang	0.257	0.107	5.005	0.429	0.062	5.005	0.469	0.096	5.005

	Melon			Bulb			Solanaceous		
	Pc	HI	MOE	Pc	HI	MOE	Pc	HI	MOE
Shanghai	0.635	0.160	2.423	0.568	0.075	3.635	0.425	0.045	3.635
Inner Mongolia	0.628	0.059	5.186	0.737	0.091	3.051	0.721	0.071	7.779
Beijing	0.300	0.079	2.054	0.730	0.232	3.081	0.200	0.053	3.081
Jilin	0.300	0.062	2.617	0.200	0.041	3.926	0.256	0.041	3.926
Sichuan	0.658	0.079	2.434	0.577	0.044	3.651	0.687	0.181	3.651
Ningxia	0.300	0.048	3.399	0.611	0.069	5.099	0.600	0.032	5.099
Guangdong	0.300	0.034	4.731	0.588	0.069	7.096	0.277	0.034	4.731
Guangxi	0.300	0.077	2.119	0.609	0.085	3.179	0.388	0.051	3.179
Jiangsu	0.528	0.055	2.968	0.256	0.036	4.452	0.449	0.042	4.452
Jiangxi	0.300	0.036	4.550	0.593	0.067	6.825	0.658	0.062	6.825
Hebei	0.412	0.047	3.452	0.729	0.130	5.177	0.672	0.070	5.177
Henan	0.477	0.060	3.476	0.700	0.099	5.214	0.513	0.047	5.214
Zhejiang	0.515	0.113	4.392	0.658	0.129	8.785	0.578	0.134	4.392
Hubei	0.300	0.065	2.481	0.423	0.052	3.722	0.258	0.065	3.722
Hunan	0.300	0.086	1.893	0.434	0.132	2.840	0.460	0.057	2.840
Fujian	0.300	0.054	3.017	0.638	0.064	4.525	1.141	0.036	4.525
Liaoning	0.293	0.079	2.054	0.612	0.098	6.352	0.676	0.071	6.352
Shanxi	0.300	0.047	3.467	0.672	0.115	5.200	0.690	0.128	5.200
Qinghai	0.434	0.101	2.927	0.503	0.113	4.391	0.395	0.060	4.391
Heilongjiang	0.570	0.086	3.337	0.559	0.095	5.005	0.539	0.088	5.005

	Fresh edible fungus			leafy			Brassica		
	Pc	HI	MOE	Pc	HI	MOE	Pc	HI	MOE
Shanghai	1.882	0.470	3.635	0.242	0.151	3.635	0.143	0.049	3.635
Inner Mongolia	0.639	0.075	7.779	0.255	0.086	7.779	0.092	0.031	6.224
Beijing	0.937	0.067	3.081	0.628	0.177	3.081	0.067	0.053	3.081
Jilin	0.494	0.062	3.926	0.085	0.041	3.926	0.067	0.041	3.926
Sichuan	2.180	0.423	3.651	0.598	0.428	3.651	0.067	0.044	3.651
Ningxia	0.200	0.032	5.099	0.372	0.033	5.099	0.189	0.041	5.099
Guangdong	0.300	0.034	4.731	0.146	0.034	7.096	0.092	0.032	4.730
Guangxi	0.200	0.051	3.179	0.686	0.051	3.179	0.067	0.051	3.179
Jiangsu	0.947	0.165	4.452	0.207	0.078	4.452	0.086	0.040	4.452
Jiangxi	0.265	0.034	6.825	0.244	0.095	6.825	0.491	0.097	6.825
Hebei	0.269	0.047	5.177	0.384	0.113	5.177	0.271	0.153	5.177
Henan	1.509	0.208	5.214	0.268	0.126	5.214	0.162	0.047	5.214
Zhejiang	1.350	0.230	4.392	0.226	0.162	4.392	0.194	0.132	4.392
Hubei	0.912	0.044	3.722	0.384	0.108	3.722	0.404	0.201	3.722
Hunan	0.460	0.057	2.840	0.223	0.057	2.840	0.138	0.122	3.840
Fujian	0.200	0.036	4.525	0.435	0.184	4.525	0.203	0.036	4.525
Liaoning	1.167	0.157	6.352	0.633	0.163	6.352	0.223	0.099	6.352
Shanxi	3.007	0.317	2.419	0.486	0.218	2.568	0.476	0.113	5.200
Qinghai	0.340	0.069	4.391	0.334	0.169	4.391	0.218	0.096	4.391
Heilongjiang	0.574	0.108	5.005	0.386	0.126	5.005	0.067	0.032	5.005

#### 3.2.1 Nemerow integrated pollution index

According to Table 3, the lead pollution of bean sprouts (2.4571) in the Guangdong Province, and edible fresh fungus (3.0065, 2.1803) in the Shaanxi Province and Sichuan Province was relatively serious. The main contaminated vegetables were fresh edible fungus, root and potato vegetables, bean sprouts and eggplant, and fruit vegetables, of which fresh edible fungus accounted for 60% of the total number of polluted combinations.

#### 3.2.2 Hazard index

The lead risk of noncarcinogenic dietary intake in all provinces/cities was extremely low, and the highest HI was only 0.4705. Therefore, the lead risk of noncarcinogenic dietary intake by eating vegetables was acceptable.

#### 3.2.3 Margin exposure

The risk of chronic dietary intake in all provinces/cities was extremely low, and the highest average daily exposure was melon vegetables in Hunan Province (0.317 μg/kg bw), MOE is 1.8931. Therefore, the lead risk of chronic dietary intake by eating vegetables is acceptable.

### 3.3 Risk classification results based on spectral clustering algorithm

The parameters in the algorithm were selected from 1 to 10, and the number of clustering categories was selected from 3 to 7. Table 4 showed the scores of some combinations. According to Table 4, the combination with the highest score was 293 points with 5 parameters and 5 cluster categories. Therefore, the risk level of dietary lead intake in vegetables was divided into five levels.

**TABLE 4:** Scores of combination with different parameters‐number of clustering categories (part)

Parameter	Cluster category number	Scores
1	3	270
1	4	276
1	5	282
1	6	97
1	7	256
5	3	127
5	4	283
5	5	293
5	6	286
5	7	289
10	3	127
10	4	92
10	5	292
10	6	246
10	7	253

The spectral clustering algorithm was used to determine the risk classification model of lead dietary intake in vegetables, the risk classification results of various vegetable–province combinations were obtained, as shown in Table 5. The combinations with risk levels above level 3 were sorted in descending order, as shown in Table 6.

**TABLE 5:** Risk classification results of vegetables in provinces

Provinces	Leafy	Root and potato	Melon	Brassica	Solanaceous	Legume	Bean sprouts	Fresh edible fungus	Bulb
Shanghai	1	1	2	1	1	1	2	5	2
Inner Mongolia	1	2	2	1	2	1	1	2	2
Beijing	2	1	1	1	1	1	1	2	2
Jilin	1	2	1	1	1	1	1	1	1
Sichuan	2	3	2	1	2	2	2	5	2
Ningxia	1	1	1	1	2	2	2	1	2
Guangdong	1	1	1	1	1	1	4	1	2
Guangxi	2	1	1	1	1	1	1	1	2
Jiangsu	1	1	2	1	1	1	1	3	1
Jiangxi	1	1	1	1	2	1	1	1	2
Hebei	1	1	1	1	2	1	1	1	2
Henan	1	1	1	1	2	1	1	3	2
Zhejiang	1	2	2	1	2	1	2	3	2
Hubei	1	1	2	1	1	1	1	3	1
Hunan	1	1	3	1	2	1	2	1	2
Fujian	1	3	1	1	3	1	1	1	2
Liaoning	2	1	1	1	2	1	1	3	1
Shanxi	2	2	1	1	2	1	1	5	2
Qinghai	1	1	2	1	1	1	2	1	1
Heilongjiang	1	1	2	1	2	1	1	2	2

**TABLE 6:** The combination of vegetable–province with a higher risk level

Vegetable categories	Province	Risk level
Fresh edible fungus	Shanxi	5
Fresh edible fungus	Sichuan	5
Fresh edible fungus	Shanghai	5
Bean sprouts	Guangdong	4
Fresh edible fungus	Henan	3
Fresh edible fungus	Zhejiang	3
Root and potato	Fujian	3
Solanaceous	Fujian	3
Fresh edible fungus	Liaoning	3
Melon	Hunan	3
Root and potato	Sichuan	3
Fresh edible fungus	Jiangsu	3
Fresh edible fungus	Hubei	3

As shown in Table 5, the combination of vegetables–province with the risk level of 1 and 2 accounted for 92.78% of the total, and that with the risk level of 4 and 5 (high‐risk level) accounted for 2.22%. Table 6 showed that the high‐risk combinations were fresh edible fungus–Shaanxi, fresh edible fungus–Sichuan, fresh edible fungus–Shanghai and bean sprouts–Guangdong. Vegetables with relatively high risk were fresh edible fungus, bean sprouts, roots, and potato vegetables.

## 4 DISCUSSION

The results of dietary exposure showed that the high and medium lead exposure of residents in all provinces/cities through vegetables were lower than the corresponding reference dose or benchmark dose; most of them were more than 5% of the reference dose, and some exceeded 20% of the reference dose. According to the relevant regulations of the Codex Alimentarius Commission (CAC), this condition was identified as significantly contributing to total exposure and needed to be managed by limiting criteria.

On the basis of exposure assessment, the risk classification model established in this study used a spectral clustering algorithm to realize the risk classification of lead pollution in vegetables. For the three indexes in the model, HI was used to characterize the risk of noncarcinogenic dietary intake in dietary exposure assessment, and MOE was used to characterize the risk of chronic dietary intake. Combined with the use of NIPI, the comprehensive evaluation needs of risk management were taken into account to a certain extent. Besides, the spectral clustering algorithm was used in the model to directly obtain the optimal classification results, in which manual selection of parameters and risk levels were not required. However, the classification results only reflected the relative risk of health hazards caused by corresponding pollutants and consumption levels in various regions, that is, the results were based on the mutual comparison between food and regional combinations and cannot reflect the absolute risk. Therefore, the combinations at the risk levels of 3, 4, and 5 mainly indicated the priority of attention.

This study mainly focused on the establishment of a risk classification model for lead pollution. If the proposed model was applied to other pollutants, such as cadmium (Cd) and chromium (Cr), the risk index of chronic dietary intake can be replaced with the index of target cancer risk (TCR).

The model developed in this study was applied to analyze the monitoring data of lead in vegetables in China in 2019, and 180 provinces/cities–vegetable combinations were ranked in terms of risk. The results were generally consistent with those obtained using classical assessment methods. A study on the levels of eight heavy metals and health risk assessment considering food consumption by China's residents based on the fifth Total Diet Study showed that the lead risk was higher in Liaoning, Shaanxi, and Sichuan provinces, and the main dietary source were vegetables, which was consistent with the evaluation results of this study. The results of a study on the evaluation and benchmarking of health risks of lead contamination in agricultural soils in east Yunnan showed that the exceedance rate of fresh edible fungus sites in Sichuan was higher, but there was no significant health risk to residents. There is no published report on the results of risk classification of lead in vegetables by applying risk classification methods at home and abroad. The existing evaluation of heavy metals in vegetables mostly adopts the contamination index method, which focuses on the comprehensive evaluation of the contamination level without considering the influence of consumption.

Food safety is a complex problem, and there is no general risk classification method for food safety. A suitable model should be established according to the classification purpose and the feasibility of the data. To reduce the influence of subjective factors and obtain scientific and effective results, objective data were used as the classification index in establishing the risk classification model in this study, and the clustering algorithm was employed to automatically select the optimal risk classification in a data‐driven way. In actual food supervision, the order of the management priority is affected by many factors. The results of risk classification can provide a basis for regulators to set management priorities based on health risks, but food safety management cannot be carried out simply according to a model or formula (Batz et al., 2004). In this study, the risk assessment and classification methods were applied by using the sampling data of vegetables in China. The results showed that the health risks of lead in vegetables can be successfully classified. The results showed that the health risks of lead in vegetables can be reliably classified. The proposed model can be used for the risk classification of various types of hazardous substances in various foods at the provincial and municipal levels, but more model applicability tests were also needed.

## CONFLICT OF INTEREST

The authors declare that they do not have any potential sources of conflict of interest.

## AUTHOR CONTRIBUTIONS

**Tong‐qiang Jiang:** Conceptualization (equal); Data curation (equal); Formal analysis (equal); Funding acquisition (equal); Investigation (equal); Methodology (equal); Project administration (equal); Resources (equal). **Zheng Wang:** Conceptualization (lead); Data curation (lead); Formal analysis (lead); Funding acquisition (lead); Investigation (lead); Methodology (lead); Project administration (lead); Resources (lead); Software (lead); Supervision (lead); Validation (lead); Visualization (lead); Writing – original draft (lead); Writing – review & editing (lead). **Qing‐chuan Zhang:** Conceptualization (equal); Data curation (equal); Resources (equal). **Zu‐zheng Wang:** Software (equal). **Bao‐lian Cheng:** Writing – review & editing (equal).

## ETHICAL APPROVAL

This study does not involve any human or animal testing. This study was Prevention and conducted in accordance with Helsinki's Declaration.

## INFORMED CONSENT

Written informed consent was obtained from all study participants.
